# MAGNET: A web-based application for gene set enrichment analysis using macrophage data sets

**DOI:** 10.1371/journal.pone.0272166

**Published:** 2023-01-11

**Authors:** Shang-Yang Chen, Gaurav Gadhvi, Deborah R. Winter

**Affiliations:** Division of Rheumatology, Feinberg School of Medicine, Northwestern University, Chicago, IL, United States of America; University of Pittsburgh, UNITED STATES

## Abstract

Characterization of gene lists obtained from high-throughput genomic experiments is an essential task to uncover the underlying biological insights. A common strategy is to perform enrichment analyses that utilize standardized biological annotations, such as GO and KEGG pathways, which attempt to encompass all domains of biology. However, this approach provides generalized, static results that may fail to capture subtleties associated with research questions within a specific domain. Thus, there is a need for an application that can provide precise, relevant results by leveraging the latest research. We have therefore developed an interactive web application, Macrophage Annotation of Gene Network Enrichment Tool (MAGNET), for performing enrichment analyses on gene sets that are specifically relevant to macrophages. Using the hypergeometric distribution, MAGNET assesses the significance of overlapping genes with annotations that were curated from published manuscripts and data repositories. We implemented numerous features that enhance utility and user-friendliness, such as the simultaneous testing of multiple gene sets, different visualization options, option to upload custom datasets, and downloadable outputs. Here, we use three example studies compared against our current database of ten publications on mouse macrophages to demonstrate that MAGNET provides relevant and unique results that complement conventional enrichment analysis tools. Although specific to macrophage datasets, we envision MAGNET will catalyze developments of similar applications in other domains of interest. MAGNET can be freely accessed at the URL https://magnet-winterlab.herokuapp.com. Website implemented in Python and PostgreSQL, with all major browsers supported. The source code is available at https://github.com/sychen9584/MAGNET.

## Introduction

Analyses of next-generation sequencing (NGS) experiments, such as RNA-seq, often produce long lists of genes as output, such as those differentially expressed between two or more conditions. It is therefore a logical and critical next step to identify the biological relevance associated with these genes. Typically, this is achieved through functional enrichment analyses that utilize standardized biological knowledge repositories, including Gene Ontology (GO) [1], Kyoto Encyclopedia of Genes and Genomes Pathways (KEGG) [2] and Molecular Signatures Database (MsigDB) [3]. These repositories annotate sets of genes with defined biological terms. These biological terms are then associated with input gene lists by calculating the overlap and performing statistical enrichment tests to assess significance. A large number of computational tools have been developed for performing this type of enrichment analysis by querying these repositories. Some of the most popular tools includes GOrilla [4], DAVID [5,6], IPA [7], and BiNGO [8]. Although these applications provide an effective way to characterize user-supplied gene lists and are now considered an essential step in typical bioinformatic workflows, they are often limited to generic results that do offer new insights to domain-specific questions. There are several reasons for this limitation: the attempt to provide terms that encompass all of biology; the static nature of the source repositories that do not account for the latest research; and the broad design of the annotation scheme.

These issues are exemplified when endeavoring to perform gene set enrichment analysis on the results of genomic experiments using macrophages. Macrophages are highly plastic immune cells that are found in virtually every tissue in health as well as having an essential role in in innate immune response [9,10]. They exhibit highly specialized functions, depending on their tissue of residence and exhibit divergent responses to environmental and pathogenic stimuli [11,12]. They have been implicated in numerous pathological models and are under active investigation as potential therapeutic targets in various diseases [13]. For this reason, their genomic landscape has been the subject of many studies across multiple biomedical disciplines [14]. However, in our experience, typical gene set enrichment analysis tools using standardized repositories with the whole genome as background primarily return generic terms related to the role of macrophages in immune response and inflammation, regardless of the context of the original experiment. Alternatively, when a set of non-differentially expressed genes is included as background to account for the macrophage transcriptome, the tools may return no significant results at all. This is because the standardized repositories do not include annotation terms to describe the novel and specialized functions of macrophages. Instead, many canonical macrophage genes are associated with the role of macrophages in innate immunity despite their relevance to other biological processes and gene pathways. The plasticity of macrophages exacerbates this limitation, but the same issue arises across domains in studies that investigate the condition-specific function of particular cell types. Thus, there is a great demand for an application that can provide precise, relevant results in accordance with the latest research.

These challenges inspired us to develop MAGNET (Macrophage Annotation of Gene Network Enrichment Tool), an interactive web application based in Python, which utilizes annotations from prior macrophage studies instead of terms from standardized biological knowledge repositories. These annotations can be curated from published manuscripts and data repositories, such as Gene Expression Omnibus (GEO) [15], that describe gene expression patterns defined by comparing macrophages across experimental conditions, such as different tissues, disease status, or time. Although a wealth of information has been published on macrophage function and identity using genomic assays, it is not always easy for a researcher outside the original study to utilize these results. MAGNET allows users to overcome the obstacles associated with retrieving individual datasets and performing bioinformatic analysis by providing a user-friendly graphical interface to compare their data with multiple other studies in parallel. By outputting the results of gene set enrichment analyses against these updated and domain-specific annotations, we show that MAGNET provides a relevant and unique characterization of user-supplied gene lists in an accessible and flexible manner.

## Methods

### Overview of MAGNET

**Fig 1:**
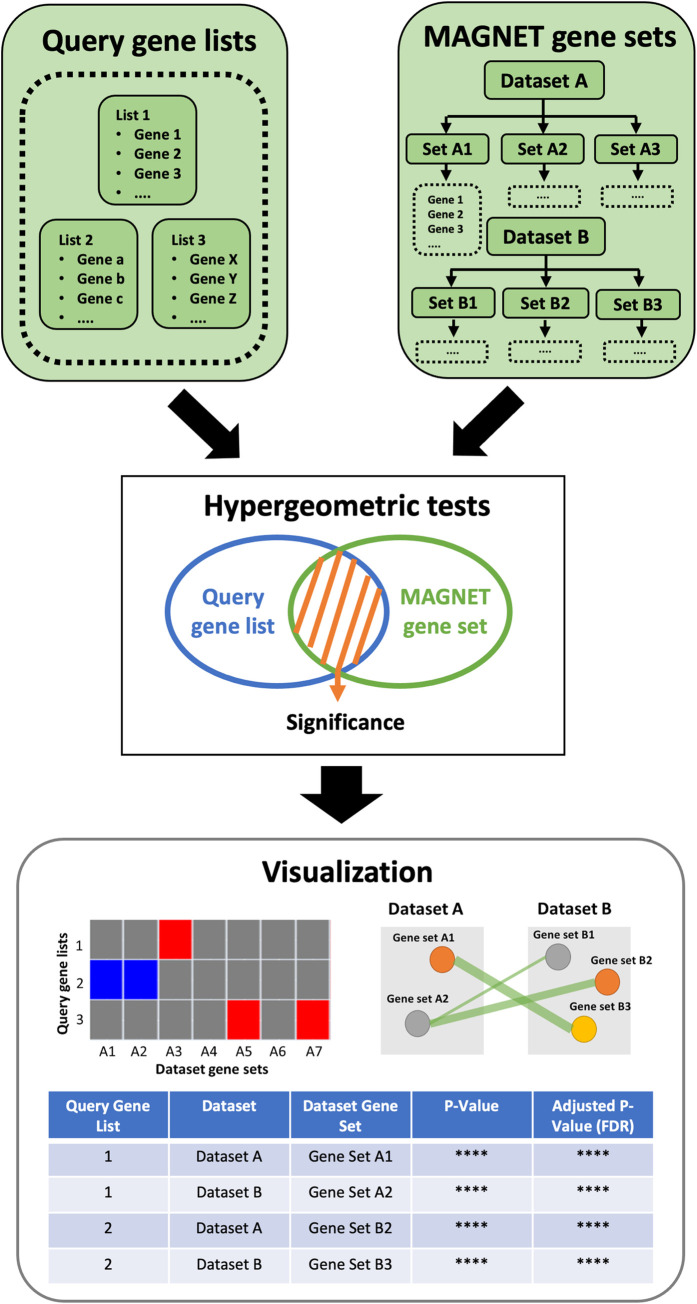
Overview schematic of MAGNET workflow. The user-supplied gene list(s) are compared with annotated gene sets that have been curated from published macrophage datasets. The significance of the overlap is assessed using the hypergeometric distribution to identify enriched (and depleted) MAGNET annotations. The results are visualized in heatmap, table, and network formats.

We developed MAGNET, a web-based interactive application for assessing and visualizing enrichments of user-supplied lists of genes against annotations curated from published literature on macrophage gene expression. The application is implemented using a Python/Django framework with PostgreSQL database integration. It is publicly available at https://magnet-winterlab.herokuapp.com. The overview schematic of MAGNET is shown in **Fig 1**. The user must input at least one gene list to query as well as a second gene list specifying the background set. Typically, we would expect the lists to originate from an RNA-seq or similar experiment, but it is not necessary. The overlap of the query gene list with MAGNET’s annotations is then compared against the overlap with the background gene set using the hypergeometric distribution. The significant annotations are visualized in multiple formats–heatmap(s) of enrichment/depletion, table of significantly enriched results, and network of intersecting annotations–to facilitate user interpretation of results.

### MAGNET annotations and database design

MAGNET consists of a curated database, in which genes are assigned to one or more annotations based on published datasets. Currently, the database incorporates ten publications on murine macrophages that encompass a variety of experimental conditions, including different tissue environments, developmental stages, and disease models (**Table 1**). These studies were chosen based on their suitability for inclusion in MAGNET but do not represent all possible datasets (**S1 Fig**). We have not performed a re-analysis of the underlying expression data. Instead, we rely on the gene sets originally reported in the manuscript or repository. We plan to continuously update and expand the database with newer relevant datasets. The database is implemented through integration of PostgreSQL and Django ORM framework. The annotations are stored as three SQL tables: Datasets, Gene Sets, and Genes. Each Dataset consists of several Gene Sets. For example, Lavin et al., 2014 [11] characterizes the transcriptional profiles of 7 tissue-resident macrophages populations compared with bone marrow monocytes and uses k-means clustering on 3348 differentially expressed genes to identify 11 clusters. The 3348 Genes are linked to Gene Sets defined by their cluster through foreign key relationships in the MAGNET database. These 11 Gene Sets are in turn linked to the Lavin et al., 2014 [11] Dataset. Gene records contain references to both Ensembl IDs and MGI gene symbols and are likely to be linked to Gene Sets in multiple Datasets. The gene records are directly utilized for computing the overlaps against user-supplied gene lists to determine which Gene Sets represent enriched annotations.

**Table 1:** Datasets currently included in the MAGNET database. Additional details are included in S1 Fig.

Title	Authors	Year	Journal	Summary	Citation
Tissue-resident macrophage enhancer landscapes are shaped by the local microenvironment	Lavin et al.	2014	Cell	**11 gene sets:** bulk RNA-seq on tissue-resident macrophages from 7 tissues	[11]
Gene expression profiles and transcriptional regulatory pathways underlying mouse tissue identity and diversity	Gautier et al.	2012	Nature Immunology	**4 gene sets**: microarrays on tissue-resident macrophages from 4 tissues	[16]
Transcriptional Heterogeneity and Lineage Commitment in Myeloid Progenitors	Paul et al.	2015	Cell	**19 gene sets:** single-cell RNA-seq on myeloid progenitors in bone marrow	[17]
Microglia development follows a stepwise program to regulate brain homeostasis	Matcovitch-Natan et al.	2016	Science	**7 gene sets:** bulk RNA-seq on developmental stages of microglia	[18]
Monocyte-derived alveolar macrophages drive lung fibrosis and persist in the lung over the life span	Misharin et al.	2017	Journal of Experimental Medicine	**5 gene sets:** bulk RNA-seq on monocytes and macrophages in the lung during pulmonary fibrosis	[19]
Microbiome Influences Prenatal and Adult Microglia in a Sex-Specific Manner	Thion et al.	2018	Cell	**7 gene sets:** microarray on developmental stages of microglia	[20]
Locally renewing resident synovial macrophages provide a protective barrier for the joint	Culemann et al.	2019	Nature	**7 gene sets:** single-cell RNA-seq on synovial macrophages in the arthritic joint	[21]
Mapping macrophage polarization over the myocardial infarction time continuum	Mouton et al.	2017	Basic Research in Cardiology	**3 gene sets:** bulk RNA-seq on cardiac macrophages after coronary artery ligation	[22]
Single cell RNA sequencing identifies unique inflammatory airspace macrophage subsets	Mould et al.	2019	JCI Insight	5 gene sets: single-cell RNA-seq of lung macrophages after intratracheal LPS	[23]
Niche-Specific Reprogramming of Epigenetic Landscapes Drives Myeloid Cell Diversity in Nonalcoholic Steatohepatitis	Seidman et al.	2020	Immunity	4 gene sets: bulk RNA-seq of liver macrophages and monocytes in NASH	[24]

### User interfaces

**Fig 2:**
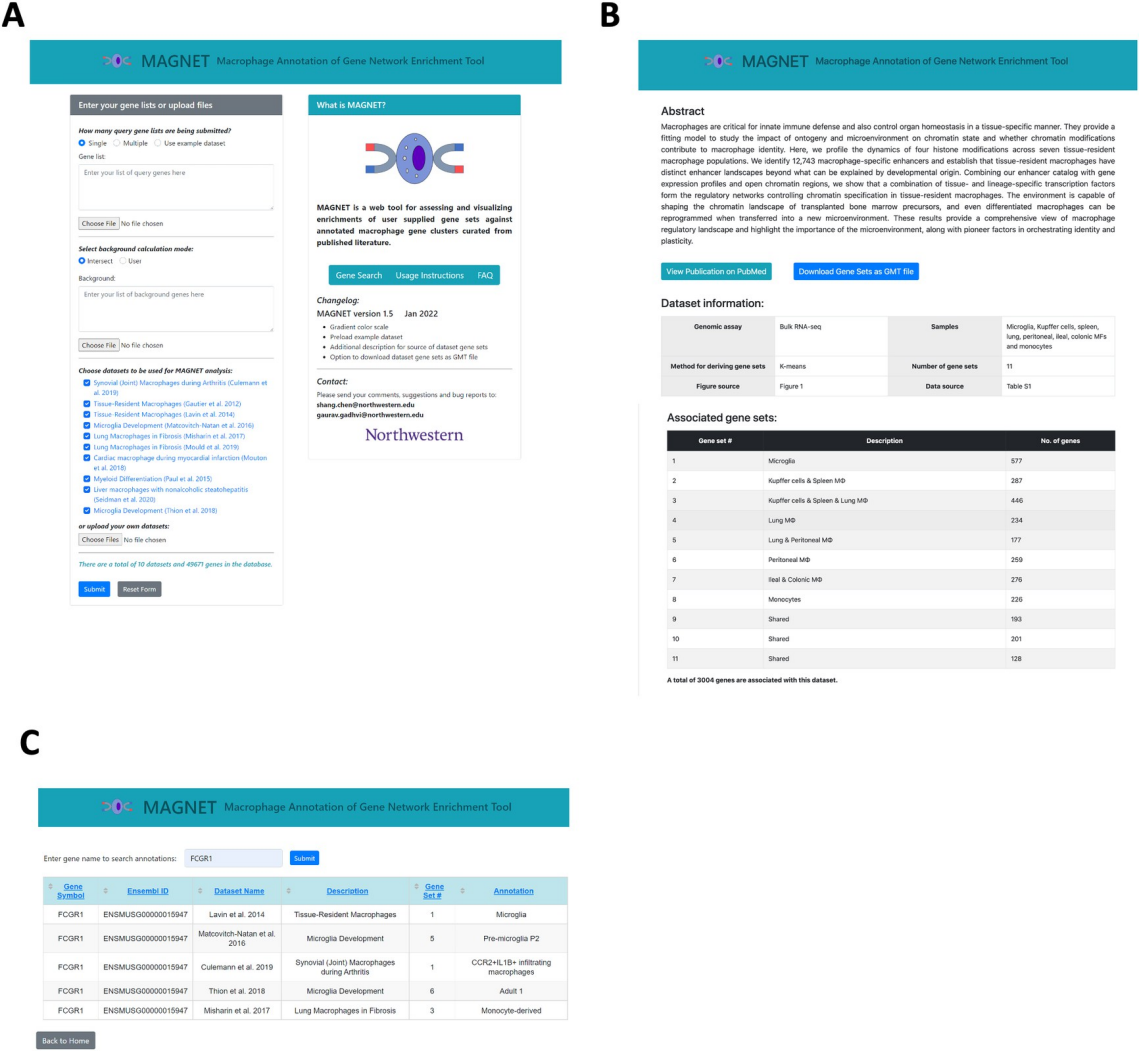
MAGNET user interfaces. **A.** Gene Set Enrichment Interface: The user is required to upload query and background gene lists, select background setting, and choose the datasets to be included in the analysis. There is also the option for the user to upload one or more custom datasets. **B.** Dataset Interface: This interface provides information on the source of each dataset and provides buttons to link to the article on Pubmed and download the GMT. Lavin et al., 2014 [11] is shown as an example. **C.** Individual Gene Interface: The user can enter genes to query against the MAGNET database of annotations. FCGR1, the gene encoding the macrophage surface marker CD64, is shown as an example.

MAGNET implements two modes of input for the query gene list (**Fig 2A**). In the traditional “single” mode, the user submits a single column of genes via the input box or file upload. This mode operates in an analogous manner to typical gene set enrichment analysis with MAGNET calculating the enrichment of the query gene list against all database annotations. In contrast, MAGNET is the first application, of which we are aware, to offer the option of multiple parallel queries. In “multiple” mode, the user can analyze several gene lists simultaneously by uploading a comma-separated file that consists of two columns: the first contains the genes while the second assigns them to different lists. The purpose of this feature is to enable the user to perform parallel enrichment analyses across related gene lists without requiring multiple queries and to visualize the results as a single output. For example, the multiple query mode is particularly useful for analyzing genes that have been clustered and provides the enrichment for each cluster independently. Regardless of the query mode, MAGNET also requires the user to input a background gene list either through the text box or a file upload. This background is part of the hypergeometric calculation described below. Finally, the user may select which curated datasets from the MAGNET database against which to perform enrichment analysis. In addition to the datasets currently included, users also have the ability to upload custom datasets against which to test their query. This original feature enables users to perform meta-analysis between any two datasets, greatly increasing the flexibility of the application.

MAGNET also includes two further interfaces for exploring datasets and genes in the database. Each curated dataset is linked to a documentation page listing essential information on the source, including the full citation information, abstract, link to PubMed, and details on the annotated gene sets (**Fig 2B**). For genes, we have implemented an interface that enables the user to query the MAGNET annotations for individual genes (**Fig 2C**). One or more genes can be entered as input and a table of the associated gene sets across all datasets is returned as output. Through this interface, the user can explore the MAGNET database without performing a full enrichment analysis.

### Hypergeometric enrichment analysis

Like other common gene set enrichment analysis tools, such as GOrilla [4] and DAVID [5,6], MAGNET utilizes the hypergeometric distribution to identify annotation terms that are significantly enriched among the query genes. MAGNET calculates the number of overlapping genes (k) independently between the user-supplied gene list(s) and each annotation. Then, the significance of that value is calculated using the hypergeometric distribution which gives the probability of observing an overlap of *k* or more genes by chance given the size of the query and annotation:

N=numberofgenesinthebackground

n=numberofgenesinthequerylist

$\begin{array}{l} K=number\;of\;genes\;in\;the\;overlap\;between\;the\;background\;and\\ \;the\;MAGNET\;annotated\;gene\;set \end{array}$

$\begin{array}{l} k=number\;of\;genes\;in\;the\;overlap\;between\;the\;query\;list\;and\\ \;the\;MAGNET\;annotated\;gene\;set \end{array}$

$$P(x\geq k)=hg(k;N,K,n)=\sum_{i=k}^{\min\left(n,K\right)}\frac{\left(C_{i}^{n})(C_{K-i}^{N-n}\right)}{C_{K}^{N}}\;where\;C_{b}^{a}=\frac{a!}{b!(a-b)!}$$

The resulting probability is reported as the p-value of enrichment. Likewise, the complementary probability (1-p), reflects the significance of depletion. Since each calculation of overlap represents a different hypothesis, MAGNET also outputs an adjusted p-value to account for multiple comparisons using the Benjamini-Hochberg method for False Discovery Rate (FDR) [25]. The total number of comparisons is the number of query gene lists multiplied by the number of annotations across all datasets.

**Fig 3:**
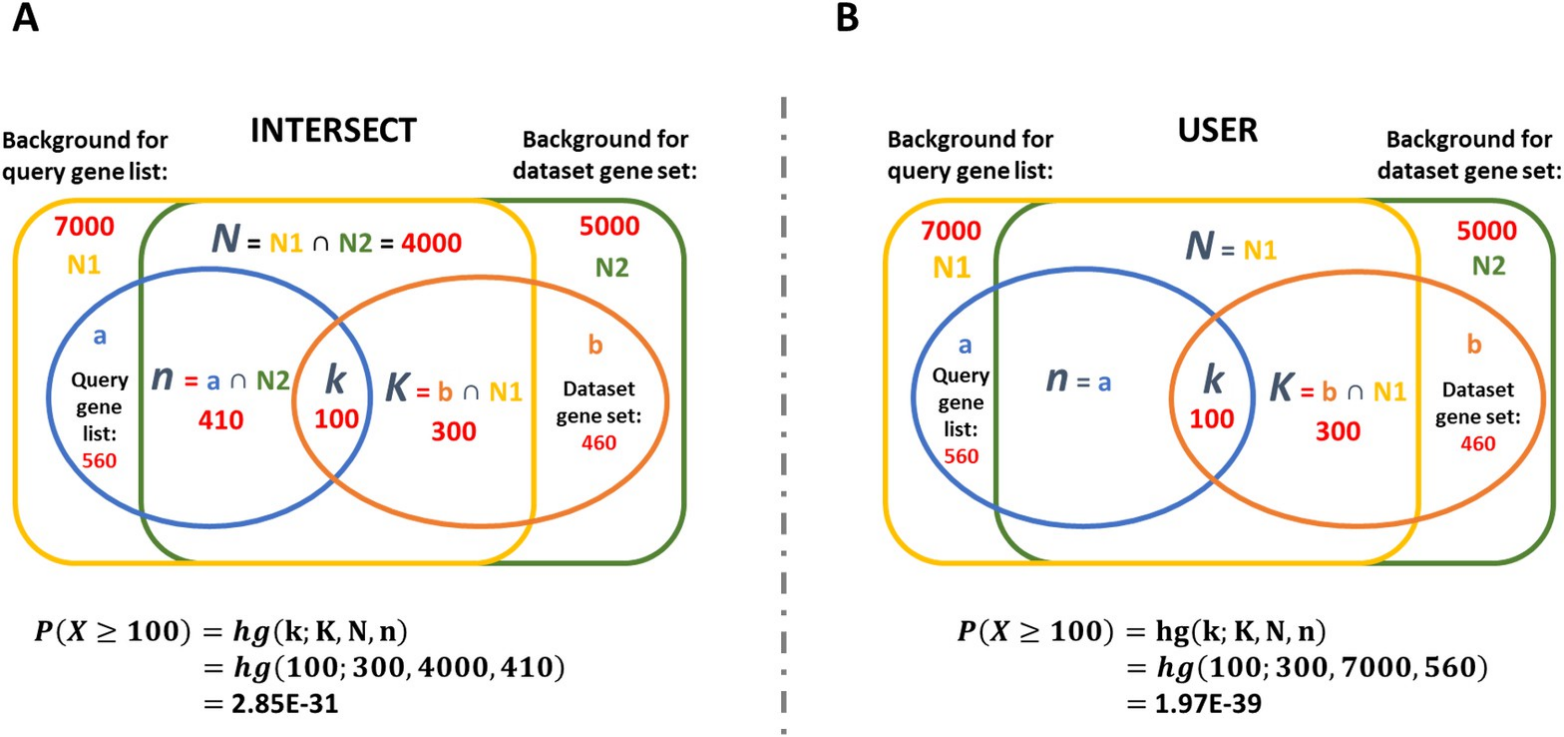
Comparison of the two settings for background in the hypergeometric test performed by MAGNET. In this example, there are 560 and 460 genes in a query gene list and annotated gene set, respectively, with an overlap of 100 between the two. There are 7000 genes in the user-supplied background with 4000 overlapping the 5000 genes from the dataset. The overlap calculations and hypergeometric p-values are illustrated for the two background settings: **A.** *INTERSECT* and **B.** *USER*.

MAGNET offers two settings for calculating the number of genes in the background, which affects the value of the parameters *N* and *n* (**Fig 3**):

1. *INTERSECT*: The background *N* is given as the intersection between the user-supplied background and the total genes in the dataset containing the annotation in question. Consequently, *n* is given as the intersection between user-supplied query list and the total dataset. This is the default setting.
2. *USER*: *N* and *n* are simply defined as the user-supplied background and query lists, respectively. This option may be preferable when there is limited overlap between the user-supplied background and the MAGNET dataset.

The hypergeometric tests are performed sequentially between the query gene list(s) and each annotated gene set from curated datasets selected or uploaded by the user.

### Visualization of results

**Fig 4:**
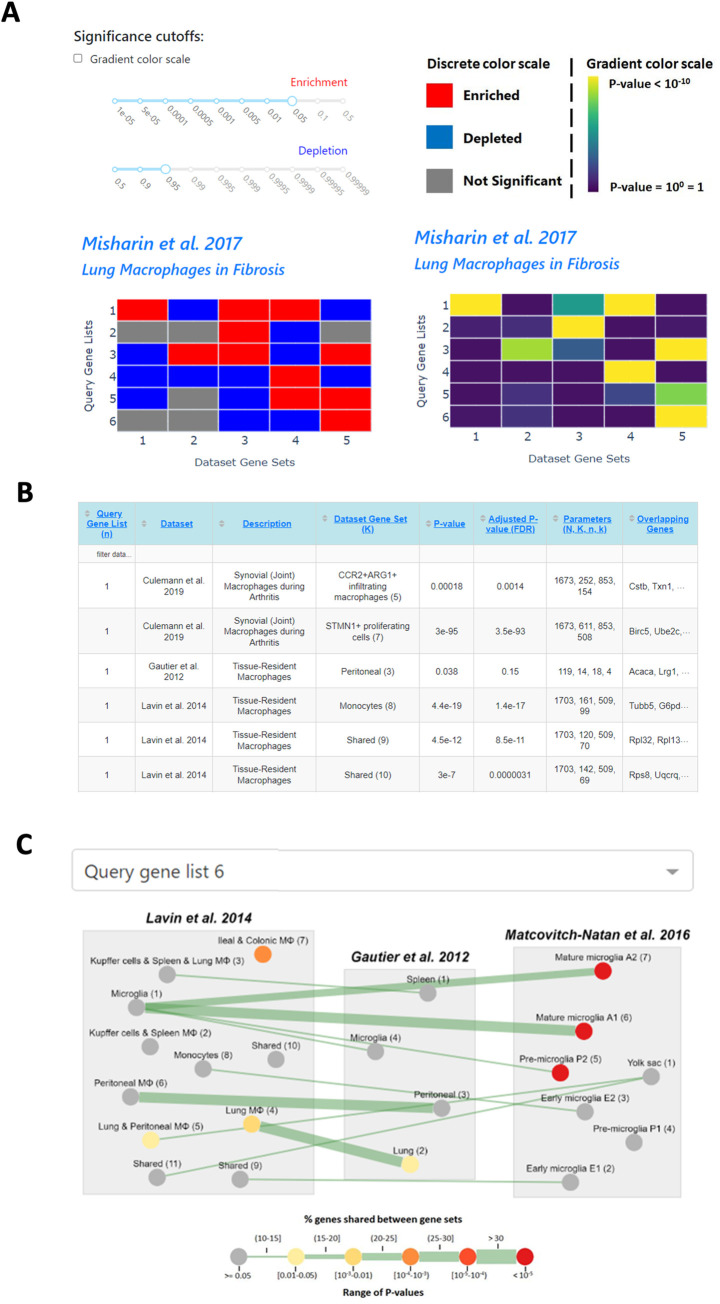
Example output of MAGNET using gene clusters reported in Koch et al., 2018 [27] as input. **A.** Heatmap output of results against curated dataset Lavin et al. 2014 [11] with p-value cutoffs of 0.05 and 0.95 for enrichment and depletion, respectively, (left) and using a gradient color scale (right). **B.** Table output of significantly enriched annotations. Only the first 10 rows of the table are shown. **C.** Network output for visualizing Cluster 1 results with the three datasets from A.

MAGNET generates three types of outputs to facilitate easy interpretation and visualization of results for the user: HEATMAP, TABLE, and NETWORK:

***HEATMAP*:** The first output consists of a heatmap corresponding to the enrichment of each dataset selected for analysis (**Fig 4A**). The heatmaps are either color-coded red or blue to reflect significant enrichments and depletions, respectively, or by a gradient color scale. Within each heatmap, the row(s) represent the query gene list(s) while the columns represent annotated gene sets from MAGNET or custom datasets. A slider widget is provided to enable the user to select their desired p-value cutoffs and update the heatmaps interactively. A mouse-over function allows the user to see additional information on the overlap, including description of the gene set, raw and adjusted p-values, and parameters used for hypergeometric test. Utilizing heatmaps to visualize significance allows the user to easily assess all the comparisons in a dataset simultaneously. Each heatmap may be downloaded separately as a png file.

***TABLE*:** The second output is a table listing all the significantly enriched gene sets (**Fig 4B**). The table includes sortable columns for the name of the source dataset, the name of the annotation, the p-value, the FDR adjusted p-value, and the parameters used in the hypergeometric calculation. The final column reports the full list of genes in the overlap. The contents in the table are updated dynamically depending on the p-value cutoff chosen. The table can be downloaded as a csv file.

***NETWORK*:** The third output is a graph depicting the network of shared genes between different MAGNET annotations with the enrichment results superimposed (**Fig 4C**). The widths of the edges correspond to the proportion of genes shared between two annotated gene sets (nodes), which are grouped by their corresponding dataset (**S2 Fig**). The underlying graph remains constant for a given choice of datasets whereas the color of the nodes changes by query to reflect the significance (p-value) of enriched annotations. The network visualization enables the user to better understand the complex relationship between different annotations. The nodes of the graph can be rearranged by the user to better visualize the edges of interest which link annotations comprising the same genes. This visualization is meant to aid with the interpretation of results to determine if enriched annotations overlap or are independent. In the multiple query mode, the user must specify for which gene list to visualize enrichment. The network visualization is generated through an interactive Cytoscape [26] plugin that enables the user to set the position of different elements. This novel method of visualizing results allows the user to interact with the data in order to appreciate the underlying complexity in the relationship of annotations across multiple datasets.

Any user-supplied query, background, or dataset genes that are not found in the MAGNET database are reported at the bottom of the output page to allow the user to identify possible errors in the gene IDs or names.

## Results

**Fig 5:**
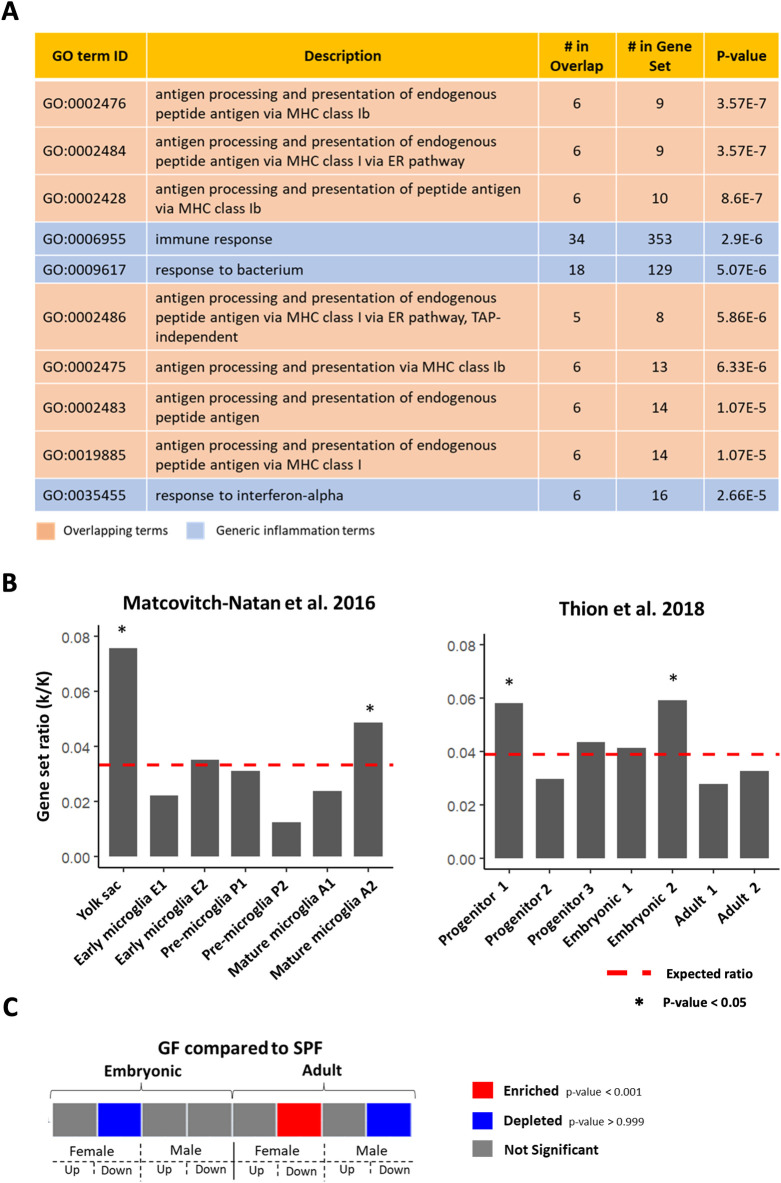
Running MAGNET with single query mode. **A.** Top 10 significant GO processes enriched for the 322 microbiome-dependent genes [18]. **B**. The ratio of genes in each annotated gene set from the *Matcovich-Natan et al*. *2016* [18] and *Thion et al*. *2018* [20] datasets that overlap the microbiome-dependent gene list as calculated by MAGNET with *INTERSECT* setting. The dashed line indicates the expected ratio based on the number of microbiome-dependent and dataset genes that overlap the user-supplied background. * indicates p-value < 0.05 for the significance of enrichment. **C.** Heatmap visualization of microbiome-dependent gene list queried against custom dataset comprising differentially expressed genes between specific pathogen free wild-type (SPF) vs. germ-free (GF) mice in male and female, embryonic and adult microglia.

To assess the performance of MAGNET on a real-world example, we used an independent RNA-seq dataset that compared gene expression of microglia–brain-resident macrophages–between mice bred with no microbiota (i.e. germ-free) and control adult mice [18]. The experiment was designed to assess the effect of the microbiome on microglia development in the brain. The original publication reported a total of 322 microbiome-dependent genes with decreased expression in germ-free mice. As reported in the original study, the top hits from GO analysis of these genes largely comprises generic biological terms associated with inflammation and overlapping terms reflecting the same small set of genes **(Fig 5A)**. Using the 7764 expressed genes documented in the publication as background, we analyzed the 322 genes as a single query with *INTERSECT* setting in MAGNET and provide the output (**S1 Table, S3 Fig**). We then summarized the results as a bar graph of enrichment (given by the gene set ratio) to compare annotations within a dataset. MAGNET reproduced the association of these genes with mature microglia genes as reported in the original publication (*Matcovitch-Natan et al. 2016 [18]- Mature microglia A2 (7),* p = 0.0292), suggesting that microglia maturity is dependent on an intact microbiome **(Fig 5B)** [18]. However, we also observed that yolk sac genes are significantly enriched among the microbiome-dependent gene list (*Matcovitch-Natan et al. 2016 [18]- Yolk sac (1),* p = 0.0001): this novel result may indicate another means by which microglia development is perturbed in germ-free mice. We next compared these results with MAGNET annotations stemming from a second more recent study of microglia development (*Thion et al. 2018 [20]*) and find that the *Progenitor 1 (1)* (p = 0.0200) and *Embryonic 2 (5)* (p = 0.0007) genes sets are enriched rather than either adult gene set (**Fig 5B**) [20]. While these results appear contradictory to the prior publication, there is room for interpretation as the gene sets do not entirely line up between datasets **(S4A Fig)**. In addition, we ran MAGNET with the INTERSECT setting to query the enrichment specifically across microglia development genes, but we observed similar results when MAGNET was run with the wider gene context in USER setting (**S4B Fig).** Aside from minor technical differences, the likely explanation for the discrepancy between datasets is the difference in the sex of the mice from each study: Matcovich-Natan et al. used only female mice while Thion et al. used both males and females. Thus, the user may infer that sex affects the role of the microbiome in microglia development. This hypothesis is supported by re-running MAGNET on this query with a custom dataset from the latter study (**Fig 5C**). We find that the microbiota-dependent genes are only enriched in adult female mice, not males, demonstrating that loss of microbiota disproportionately perturbs the microglial phenotype of female mice. This example demonstrates the utility of MAGNET in providing meaningful results reflecting the complexity of biology despite the possibility of divergent results. By simplifying comparison of user-supplied gene lists with published datasets, MAGNET enables further insight into macrophage biology.

**Fig 6:**
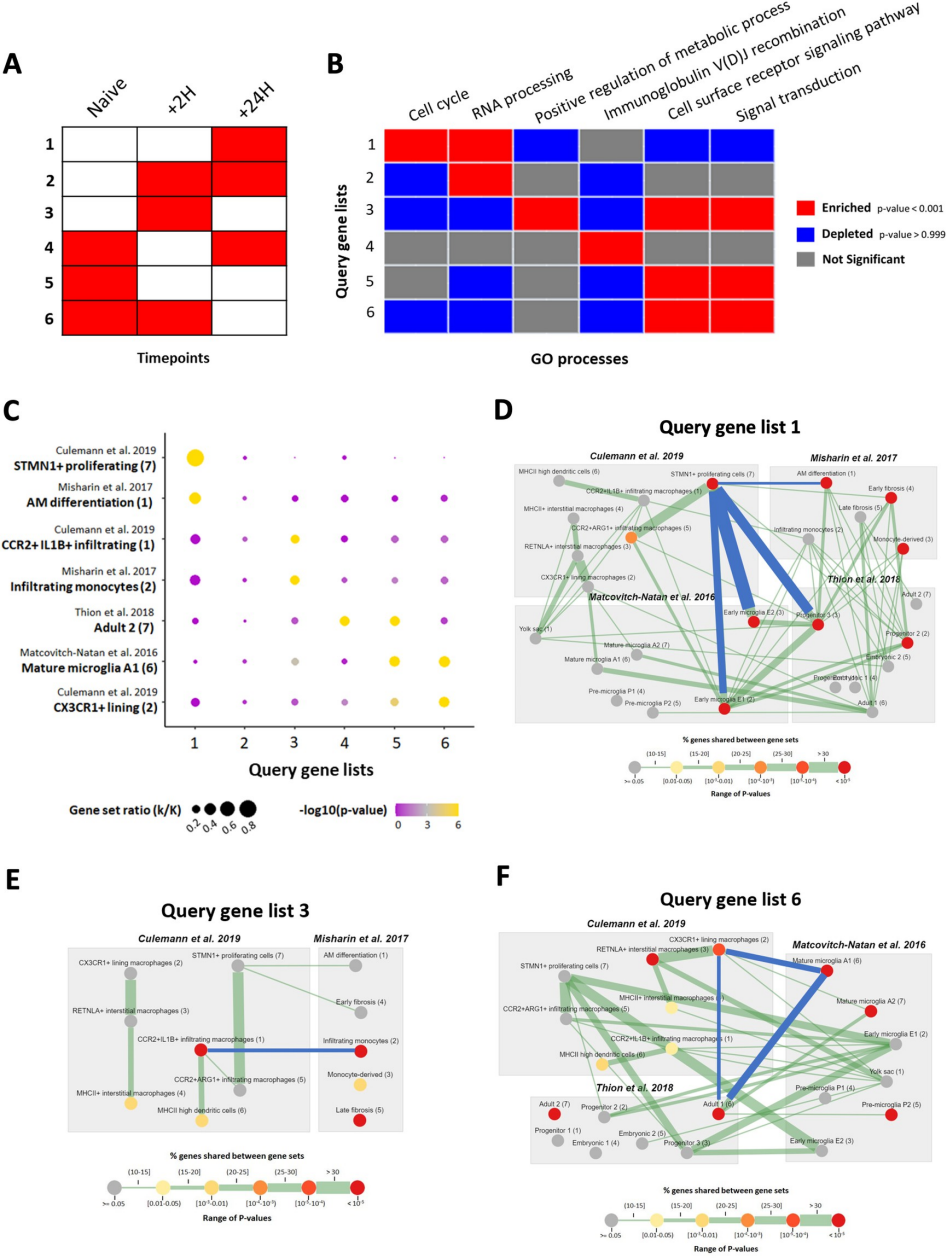
Running MAGNET with multiple query mode. **A.** Schematic of the 6 gene expression clusters defined in Koch et al. 2018 [27]. Red in each row indicates the cluster(s) with the highest relative expression. **B.** Heatmap visualization from MAGNET showing enrichment of GO processes across the 6 clusters from Koch et al. 2018 [27] using a custom user dataset with *USER* setting [27]. **C.** Enrichment of select MAGNET gene sets across the 6 clusters from Koch et al. 2018 [27] with *INTERSECT* setting. The color scale reflects p-values (-log10) whereas the size represents the ratio of genes in the annotated gene set that overlap each cluster. **D.** Network visualization of Query Gene List (Cluster) 1 enriched annotations. **E.** Network visualization of Query Gene List (Cluster) 3 enriched annotations. **F.** Network visualization of Query Gene List (Cluster) 6 enriched annotations.

Next, we tested the multiple query mode in MAGNET using another independent RNA-seq dataset that profiled gene expression in alveolar macrophages (AM) from a murine model of lung transplantation [27]. The experiment was designed to compare naïve alveolar macrophages with those isolated from newly implanted lungs at 2- and 24-hours post-reperfusion. The processed dataset consists of 7166 differentially expressed genes that clustered into 6 different expression patterns using k-means (**Fig 6A)**. The original publication reported the top GO hit for each cluster; however, because the enrichment was calculated separately for each cluster, it is not possible to compare terms in order to build a cohesive narrative. To better understand how these processes vary across the whole dataset, we input these 6 clusters in MAGNET multiple query mode using the GO terms as custom datasets with the 7166 genes as background (**Fig 6B and S2 Table)**. We find that some of these processes are, in fact, shared across clusters. Moreover, we also input the 6 clusters in the multiple query mode of MAGNET (*INTERSECT*) using the built-in datasets and provide the output **(S5 Fig and S3 Table**). To demonstrate an alternative approach to summarizing the results, we then chose select annotations across datasets to feature as a bubble plot (**Fig 6C**). For instance, the genes that are preferentially expressed at 24 hours (Cluster 1) are highly enriched for *STMN1+ proliferating cells (7)* from the *Culemann et al. 2019 [21]* dataset *(p = 2*.*95x10*^*-95*^*)*. This is consistent with the unique enrichment of the GO process *Cell cycle* in this cluster. Other MAGNET annotations that share genes with *Culemann 7* and are associated with cell cycle in their respective datasets–such as *Thion et al. 2018 [20]—Progenitor 3 (3)* and *Matcovitch-Natan et al. 2016 [18]—Early microglia E1/E2 (2/3)–*were also enriched (**Fig 6D**). In addition, we find that Cluster 1 genes significantly overlap *Misharin et al*. *2017* [19]–*AM differentiation (p = 8*.*15x10*^*-95*^*)* which suggests that there is similarity in gene expression after transplantation and as monocytes differentiate into alveolar macrophages during fibrosis. These results fit with the current paradigm of infiltrating monocytes replacing tissue-resident macrophages following a disruption to the niche [28]. The hypothesis that the AM compartment in this dataset comprises monocyte-derived cells starting at 2 hours post-reperfusion is further supported by the enrichment of monocyte-related annotations in Cluster 3 (*Culemann et al. 2019 [21]—CCR2+IL1B+ infiltrating macrophages (1)*; *Misharin et al. 2017 [19]—Infiltrating monocytes (2)*) (**Fig 6E**). Similarly, the shift from annotations representing more mature phenotypes (*Thion et al. 2018 [20]—Adult 2 (7); Matcovitch-Natan et al. 2016 [18]—Mature microglia A1 (6)*; *Culemann et al. 2019 [21]—CX3CR1+ lining macrophages (2)*), particularly in Clusters 5 and 6, suggests that the original tissue-resident macrophages are replaced (**Fig 6F**). MAGNET also allows the user to determine how the genes in a given annotation are distributed across the query gene lists. As an example, we visualized the gene set specific to *Lung macrophages (4)* from *Lavin et al. 2014 [11]* and found that nearly half of the genes that overlap this dataset are in Clusters 5 and 6 (**S6A Fig**). Specific genes can then be further investigated using the Individual Gene Interface, such as Car4 in Cluster1, Cd9 in Cluster 5, and Ear2 in Cluster 6 (**S6B Fig**). Finally, to demonstrate the utility of MAGNET on a query with nothing in common with the current datasets, we used gene lists from a publication on tumor-associated macrophages in breast cancer models [29]. We found that MAGNET outputs distinct enrichments for each gene list that are consistent with the underlying biology and meaningfully supplement the GO results reported in the original publication (**S4 Table**). For example, KEP-TAM were reported to be associated with generic metabolism processes, but we found that those genes are also enriched for annotations linked to infiltrating monocytes (**S7 Fig**). Taken together, we demonstrated that MAGNET’s multiple query mode enables parallel comparison of gene lists to expand conventional enrichment results by leveraging the latest macrophage research for a more complete picture of the underlying biology.

## Discussion

The explosion of high-throughput genomic data has facilitated rapid developments in bioinformatic tools for gene set enrichment analysis in recent decades. The majority of available enrichment tools aim to be all-encompassing in order to maximize the ability to handle data across the full range of different biological experiments. However, given the breadth and depth of biological domains, it is difficult to achieve this goal and still return results that are relevant to specific questions. In macrophage biology, this issue is exacerbated as macrophages demonstrate an astounding plasticity and play a role in multiple developmental, homeostatic, and disease processes [10,13]. The inability of conventional enrichment tools to capture this variability is illustrated by the preponderance of generic terms among the results. For example, many contemporary studies focus on interrogating macrophage heterogeneity within a tissue in a disease model compared with steady-state [19,21,22,24,30,31]. These studies are commonly interested in differences in ontogeny and function between macrophage subpopulations (i.e., monocyte-derived vs. tissue-resident). Thus, a tool that returns the term “inflammation” or “defense response” is unlikely to lead to meaningful biological insights. Since conventional gene set enrichment analysis is not designed for domain-specific research questions, their potential to foster cutting-edge research is limited.

We therefore designed MAGNET to address this issue by utilizing the annotated macrophage datasets available rather than standardized knowledge repositories. MAGNET represents an innovative approach to gene set enrichment analysis for domain-specific questions. Using gene lists from Matcovitch-Natan et al. 2016 [18] and Koch et al. 2018 [27] as examples, we demonstrated that MAGNET provides relevant and unique results when characterizing macrophage-related gene lists. Furthermore, MAGNET offers several novel functionalities that have not previously been incorporated into conventional enrichment tools, including multiple query mode, a gene search interface, alternate visualizations (heatmap and network), and the ability to upload custom dataset annotations. As an easily accessible online application with modifiable parameters, MAGNET is a user-friendly and flexible tool. We expect MAGNET to serve as a valuable addition to the bioinformatic workflow associated with genomic analysis in the field of macrophage biology and beyond.

Because it is based on a manually curated database, MAGNET exhibits some inherent limitations. First, the selection and availability of datasets might lead to unintentional biases in the annotations towards certain tissues, disease states, etc. This shortcoming will decline over time as more datasets become available and is already somewhat ameliorated by enabling the user to upload their own dataset(s) for meta-analysis between any two experiments. Ideally, MAGNET would cover all relevant datasets as they become available, and we plan to continuously expand the underlying database. However, manual curation means that the time required to incorporate a new dataset is the major bottleneck for scaling up the database. Inclusion in MAGNET is also dependent on data being provided in a compatible format; some datasets are more difficult to process than others or require additional information from the authors. As we continue to expand MAGNET, we will explore the possibility of automating the curation process using web scraping, with the goal of striking a balance between quality and quantity. Finally, since the value of each dataset is dependent on the experimental and analytical approach of the original study, there may be variability in the relevance of the output annotations. As demonstrated above with microglia development, different datasets may be associated with similar annotations, but result in divergent enrichment results. Thus, the user must use their best judgement to assess results and resolve any discrepancies.

We plan to continuously optimize and introduce new functionalities to MAGNET. For example, inclusion of additional metrics may improve the interpretability of enrichment results. The Jaccard index, which provides an intuitive assessment of similarity between gene sets, would extend the multiple query mode by enabling comparison of all user-supplied query gene lists against an entire MAGNET dataset. Another possibility is to output an odds ratio that, unlike p-values for which the magnitude should not be compared across tests, would provide a metric for ranking and prioritization of enriched annotations. In the current implementation, MAGNET may be used to annotate gene lists from experiments on other species by first converting genes into the murine orthologs. However, as the number of studies performed increases (for example, in humans), we intend to implement independent species-specific versions of the application. Furthermore, beyond gene sets, a similar approach based on published datasets could be used to annotate genomic regions, such as promoters and enhancers, as demonstrated by other tools [32,33]. In the larger context, the software behind MAGNET could easily be adapted to other domains where conventional gene set enrichment tools are similarly limited. We envision that the introduction of MAGNET will catalyze the development of similar and more targeted applications for different cell types, tissues, or fields, such as cancer and neurobiology.

## Supporting information

S1 Fig MAGNET Datasets. **A.** Selection criteria for choosing datasets suitable for MAGNET. **B.** Additional details on MAGNET datasets.
(TIF)

S2 Fig Network visualization of all gene sets curated in MAGNET. (TIF)

S3 Fig Heatmap visualization from MAGNET with single query mode. The results show enrichment for all current datasets in single query mode of the microbiome-dependent gene list from the *Matcovich-Natan et al. 2016 [18]* with *INTERSECT* setting.
(TIF)

S4 Fig Alternate options for MAGNET with single query mode. **A.** Network visualization from MAGNET showing enrichment of *Matcovitch-Natan et al. 2016 [18]* and *Thion et al. 2018 [20]* datasets using the query list of microbiome-dependent genes with *INTERSECT* setting**. B.** The ratio of genes in each annotated gene set from the Matcovich-Natan et al. 2016 [18] and Thion et al. 2018 [20] datasets that overlap the microbiome-dependent gene list as calculated by MAGNET with *USER* setting. The dashed line indicates the expected ratio based on the total number of microbiome-dependent and dataset genes. * indicates p-value < 0.05 for the significance of enrichment.
(TIF)

S5 Fig Heatmap visualization from MAGNET with multiple query mode. The results show enrichment for all current datasets in multiple query mode of 6 gene expression clusters from Koch et al. 2018 [27] with *INTERSECT* setting.
(TIF)

S6 Fig Overlap with Lung Macrophage genes. **A.** Distribution of genes from the *Lavin et al. 2014 [11] Lung macrophages* annotation across the 6 clusters from Koch et al. 2018 [27]. **B.** Individual Gene Interface for select genes from the *Lung Macrophage* annotation that overlap the 6 clusters from Koch et al. 2018 [27].
(TIF)

S7 Fig Select heatmap visualization from MAGNET on TAM gene lists. The results show enrichment from *Culemann et al. 2019 [21]* and *Seidman et al. 2020 [24]* in multiple query mode of 3 gene lists from Tuit et al. 2019 [29] with *INTERSECT* setting.
(TIF)

S1 Table Single query mode results on MAGNET database using microbiome-dependent genes reported in Matcovitch-Natan et al. 2016 [18] as input (*INTERSECT* setting). (CSV)

S2 Table Multiple query mode results from MAGNET on a custom user dataset using 6 gene expression clusters reported in Koch et al. 2018 [27] as input (*USER* setting). (CSV)

S3 Table Multiple query mode results on MAGNET database using 6 gene expression clusters reported in Koch et al. 2018 [27] as input (*INTERSECT* setting). (CSV)

S4 Table Multiple query mode results on MAGNET database using 3 gene set in TAMs reported in Tuit et al. 2019 [29] as input (*INTERSECT* setting). (CSV)
