# Supplementary figures and images for: MAGNET: A web-based application for gene set enrichment analysis using macrophage data sets
Source: PLoS One. 2023 Jan 11;18(1):e0272166. doi: 10.1371/journal.pone.0272166 (PMC9833518; doi:10.1371/journal.pone.0272166)

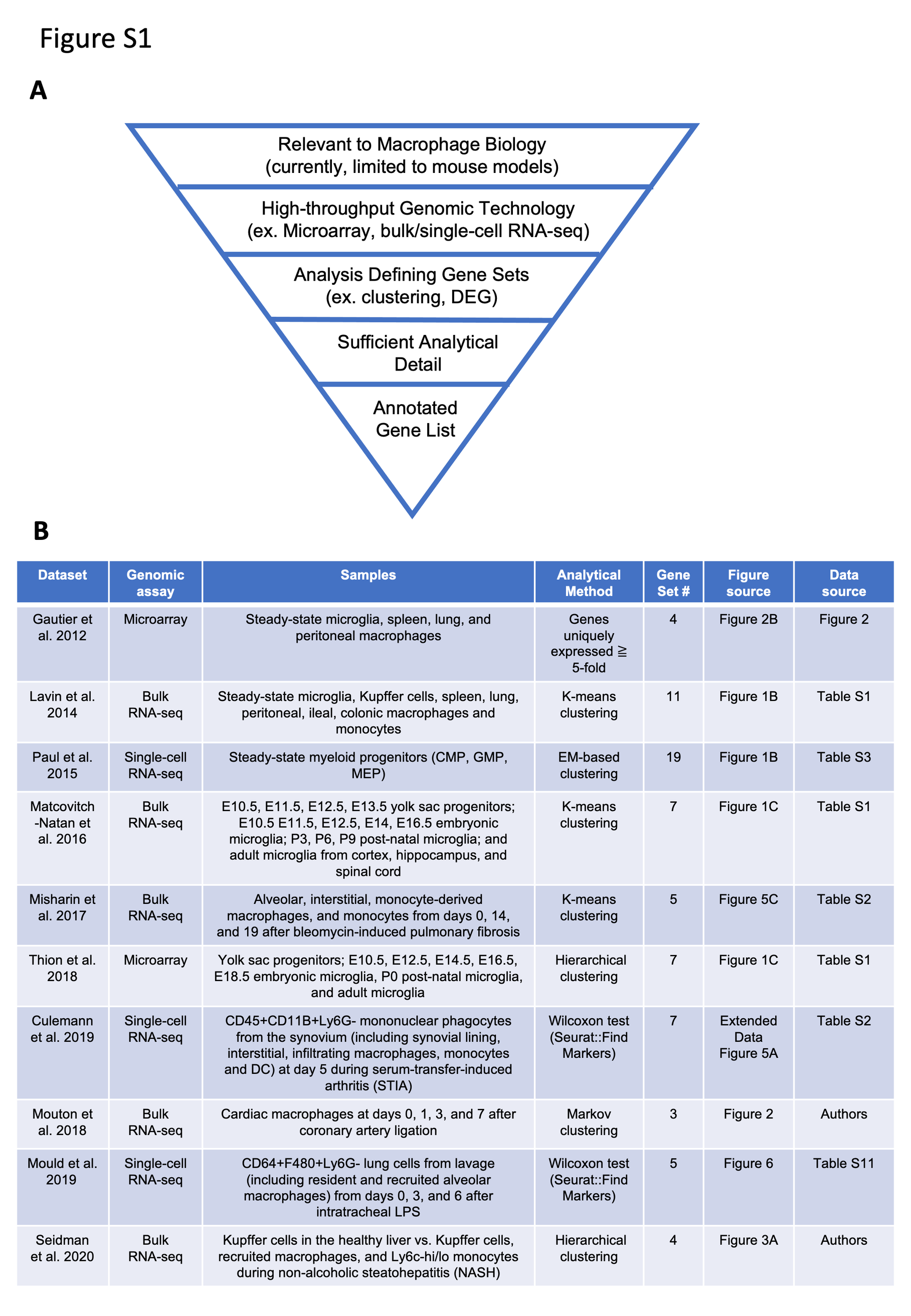

Supplement: S1 Fig — A. Selection criteria for choosing datasets suitable for MAGNET. B. Additional details on MAGNET datasets. (TIF) [file pone.0272166.s001.tif]

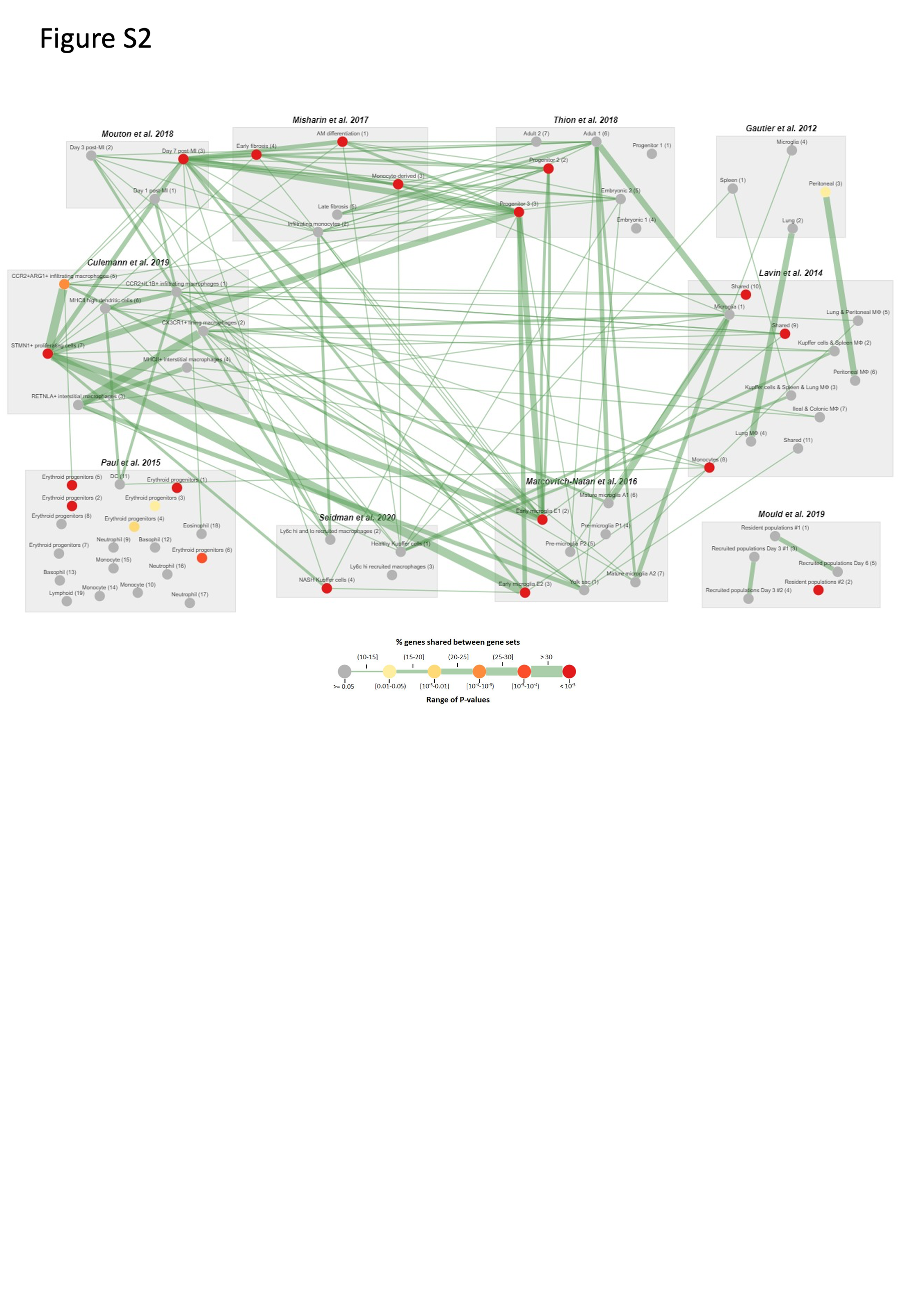

Supplement: S2 Fig — (TIF) [file pone.0272166.s002.tif]

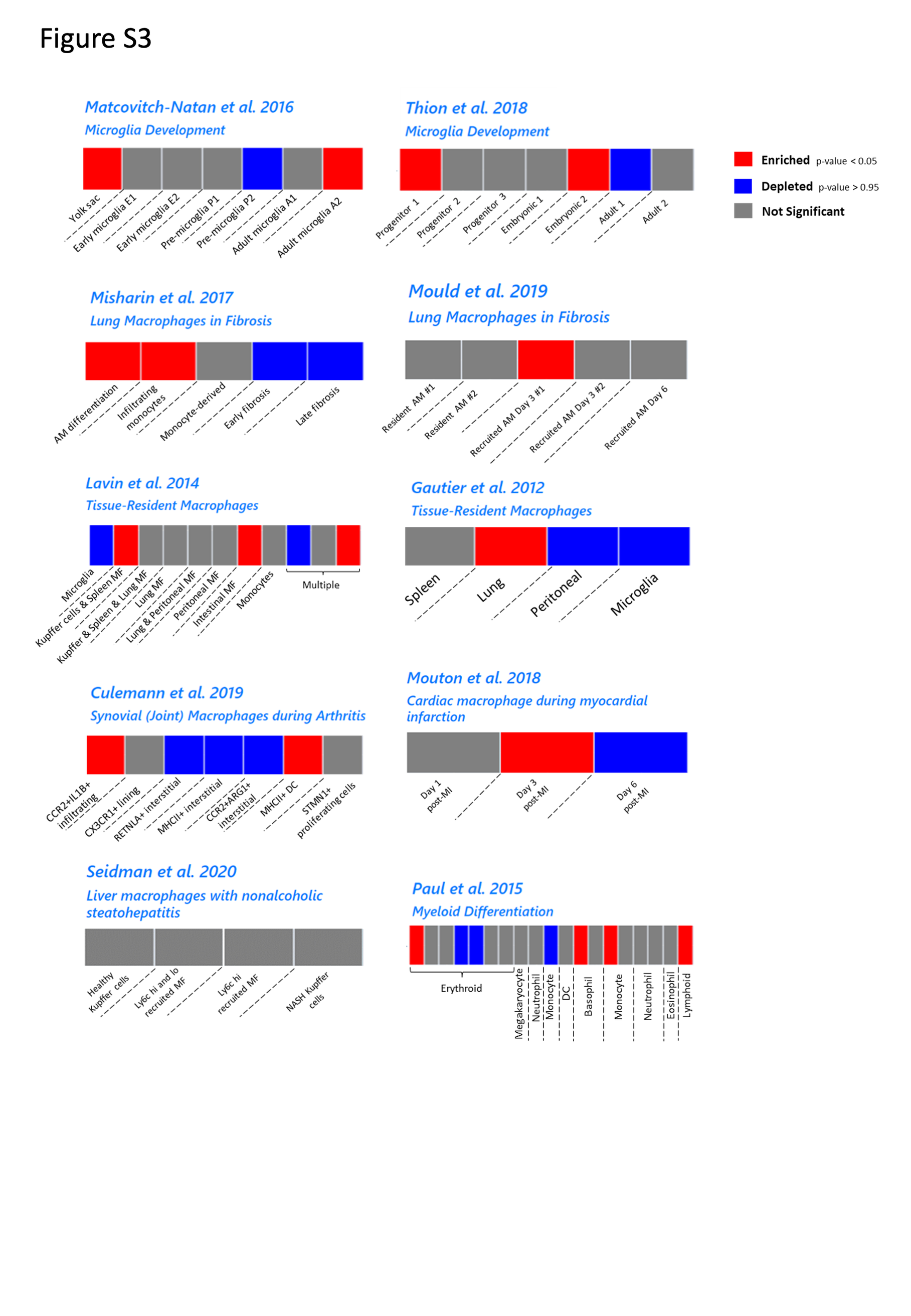

Supplement: S3 Fig — The results show enrichment for all current datasets in single query mode of the microbiome-dependent gene list from the Matcovich-Natan et al. 2016 [18] with INTERSECT setting. (TIF) [file pone.0272166.s003.tif]

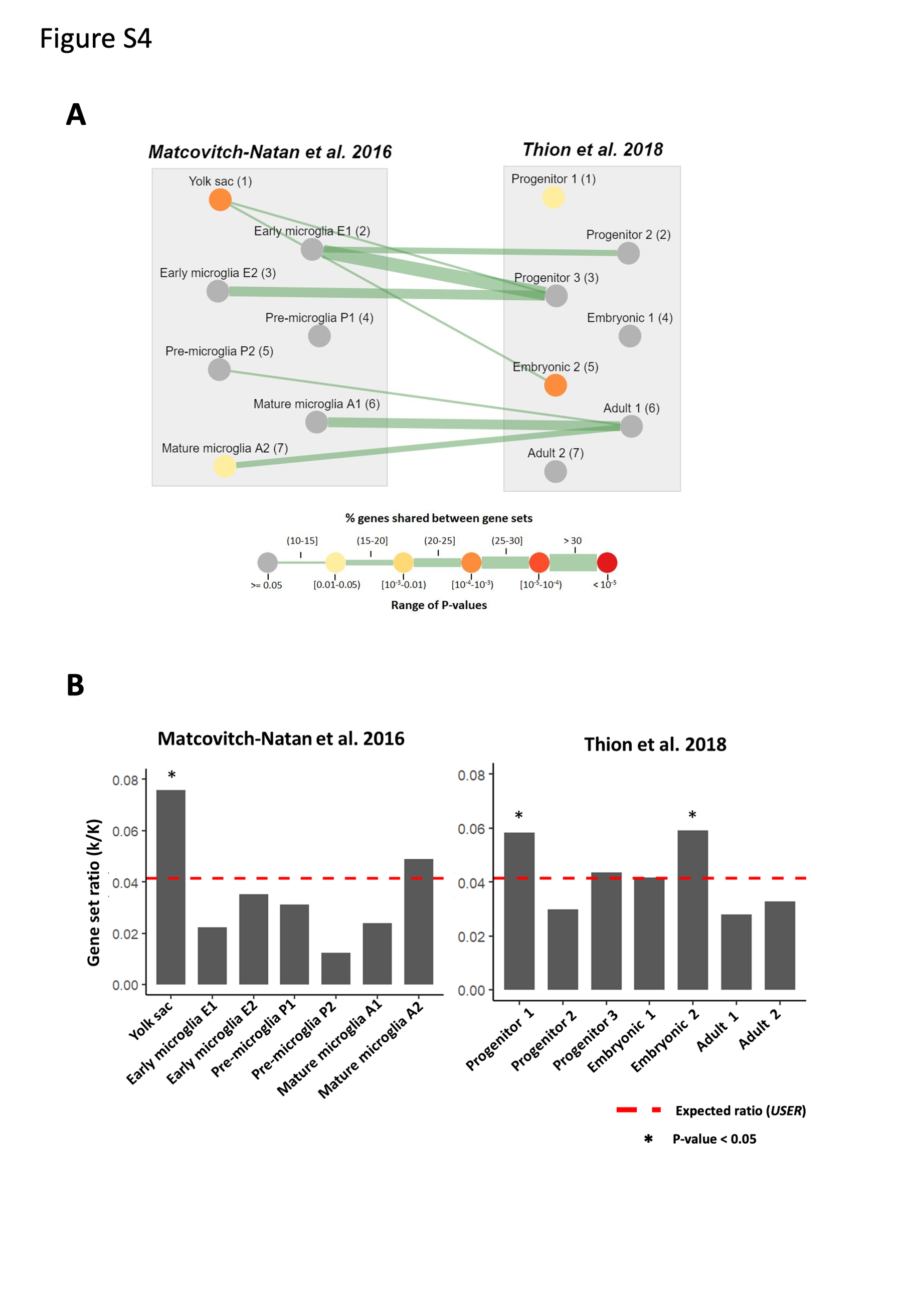

Supplement: S4 Fig — A. Network visualization from MAGNET showing enrichment of Matcovitch-Natan et al. 2016 [18] and Thion et al. 2018 [20] datasets using the query list of microbiome-dependent genes with INTERSECT setting. B. The ratio of genes in each annotated gene set from the Matcovich-Natan et al. 2016 [18] and Thion et al. 2018 [20] datasets that overlap the microbiome-dependent gene list as calculated by MAGNET with USER setting. The dashed line indicates the expected ratio based on the total number of microbiome-dependent and dataset genes. * indicates p-value < 0.05 for the significance of enrichment. (TIF) [file pone.0272166.s004.tif]

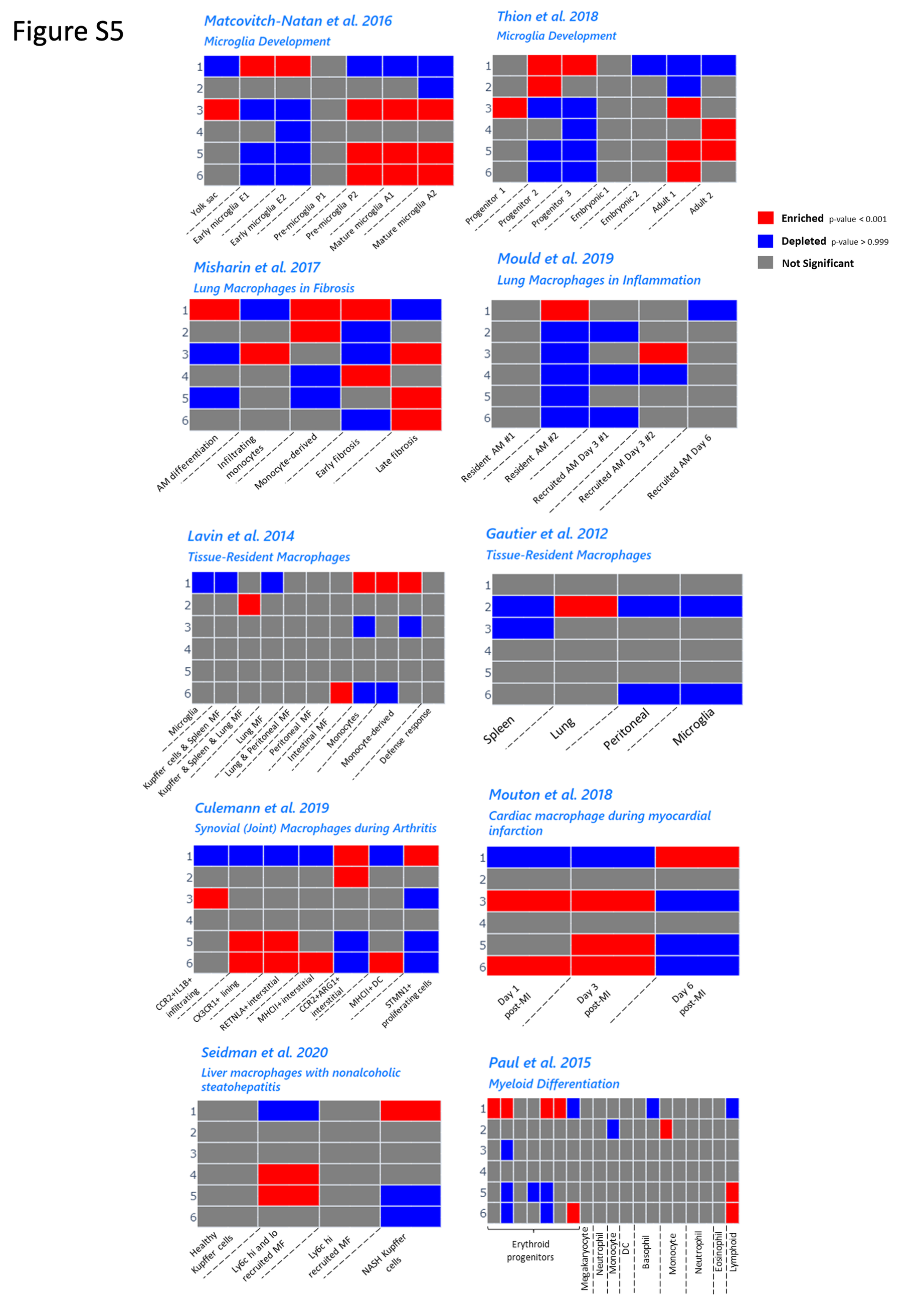

Supplement: S5 Fig — The results show enrichment for all current datasets in multiple query mode of 6 gene expression clusters from Koch et al. 2018 [27] with INTERSECT setting. (TIF) [file pone.0272166.s005.tif]

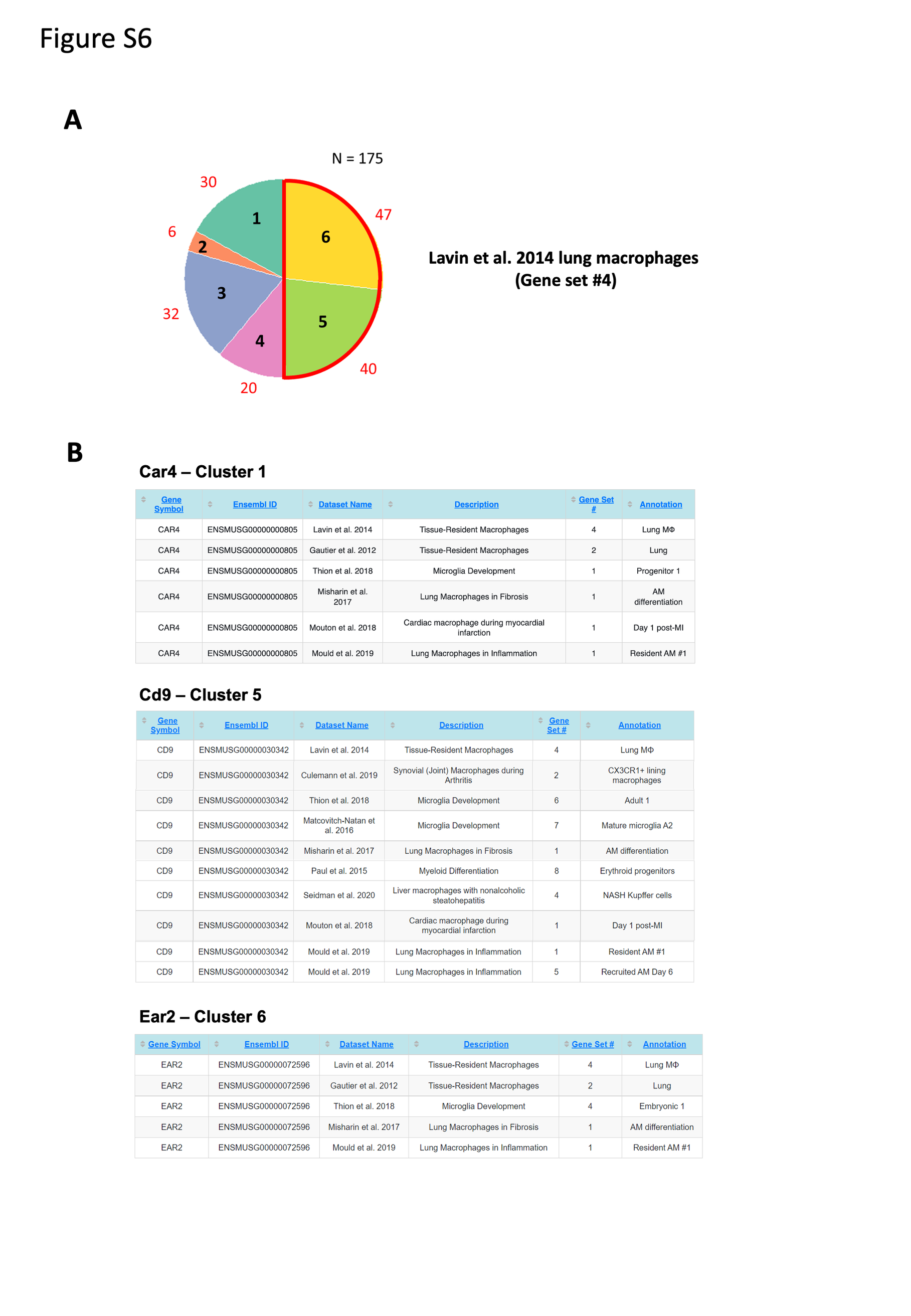

Supplement: S6 Fig — A. Distribution of genes from the Lavin et al. 2014 [11] Lung macrophages annotation across the 6 clusters from Koch et al. 2018 [27]. B. Individual Gene Interface for select genes from the Lung Macrophage annotation that overlap the 6 clusters from Koch et al. 2018 [27]. (TIF) [file pone.0272166.s006.tif]

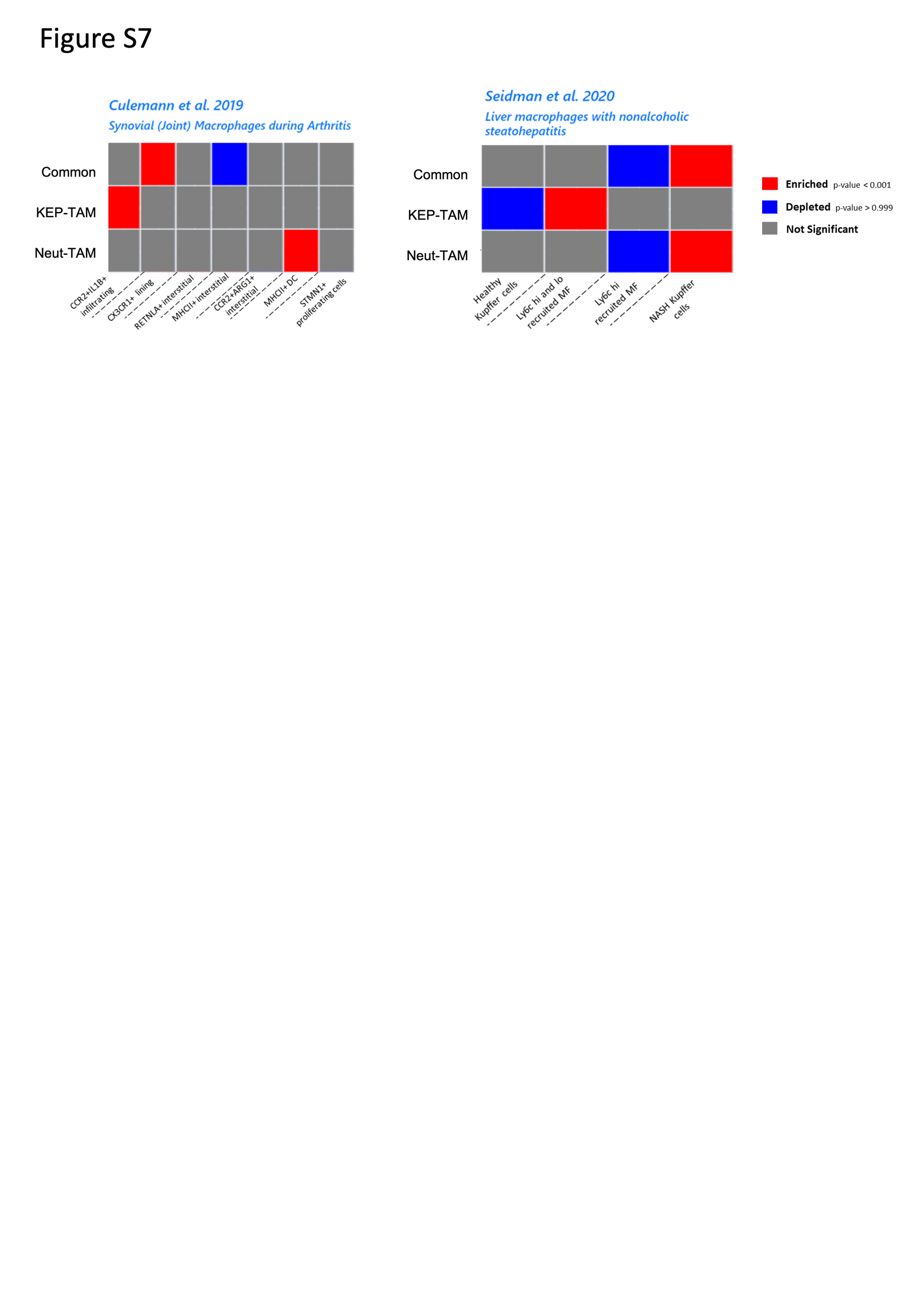

Supplement: S7 Fig — The results show enrichment from Culemann et al. 2019 [21] and Seidman et al. 2020 [24] in multiple query mode of 3 gene lists from Tuit et al. 2019 [29] with INTERSECT setting. (TIF) [file pone.0272166.s007.tif]
